# Smoking Habit in Severe Obese after bariatric procedures

**DOI:** 10.1186/s12971-015-0045-8

**Published:** 2015-07-29

**Authors:** Mauro Maniscalco, Pierluigi Carratù, Stanislao Faraone, Maria Rosaria Cerbone, Stefano Cristiano, Anna Zedda, Onofrio Resta

**Affiliations:** Section of Respiratory Medicine, Hospital S. Maria della Pietà, Casoria, Naples, Italy; Institute of Respiratory Disease, University of Medicine, Piazza G. Cesare 12, 70124 Bari, Italy; Department of Surgery, Hospital S. Maria della Pietà, Casoria, Naples, Italy

**Keywords:** Smoke, Exhaled, Surgery, Obesity

## Abstract

**Background:**

Bariatric procedures provide an effective means of short term weight loss and sustained weight control for the morbidly obese. The effect of bariatric procedures on smoking habit in obese subjects is not well known. Therefore, we examined the short term effect of bariatric surgery on smoking habit of severe obese patients up to 12 months from the intervention.

**Patients and Methods:**

Smoking habit was assessed in a cohort of 78 morbid smoking obese patients followed at our clinic for bariatric procedures. They underwent non surgical intra-gastric balloon (IB) or surgical procedures such as lap–band laparoscopic surgery (LAGB) or sleeve gastrectomy/gastric by-pass (SPG). Subjects were administered a written questionnaire about their smoking habit before and 3, 6 and 12 months after the procedures.

**Results:**

No differences were found among the three groups at 6 and 12 months after the procedures (IB 21 %, LAGB 6 %, SPG 5 %; and IB 14 %, LAGB 3 %, SPG 5 %). Only after 3 months, the rate of quitting of the IB group was higher than LAGB and SPG groups (36 %, 6 % and 5 %, respectively; *p* = 0.02).

**Conclusions:**

Bariatric procedures have no effects on smoking habit of moderate-to-heavy smoker severe obese patients. The use of other traditional smoking cessation methods in patients undergone to bariatric procedures should be implemented.

## Introduction

Obesity has grown to epidemic proportions in western societies and is associated with considerable increase in morbidity and mortality [1]. Although obese patients may be heavy smokers up to 40 % [2], they are not receiving specific attention about smoking cessation [3]. Furthermore, most programs for stopping smoking do not encourage simultaneous weight control because changing several health behaviors has not been very successful [4].

Bariatric procedures for obesity are effective means of weight loss and sustained weight control for the morbidly obese. The most commonly performed invasive procedures are Roux-en-Y gastric bypass, sleeve gastrectomy, and bilio-pancreatic diversion. Less complex procedures to treat obesity are intra-gastric balloons (IB) and lap–band laparoscopic surgery (LAGB). They are respectively a temporary non-surgical and a surgical obesity treatment that induce short-term weight loss by partially filling or restricting the size of the stomach to achieve satiety and reduce the amount of food intake [5].

Only few studies have investigated the smoking status in obese patients before and after bariatric procedures, only examining patients after invasive procedures such as gastric bypass [6, 7], without studying patients after less invasive procedures such as IB.

The aim of our study was to prospectively examine the effect of different bariatric procedures (IB, LAGB, sleeve gastrectomy and laparoscopic gastric bypass) on smoking habit of severe obese.

## Materials and methods

### Participants

This is a prospective, longitudinal study involving a cohort of 78 smoking morbid obese subjects, followed at our clinic. They underwent bariatric surgery between January 2008 and December 2011. We included patients with a minimum body mass index (BMI) of 35 (calculated as weight in kilograms divided by height in meters squared), with a stable weight for at least five years. Patients who smoked less than 10 cigarettes per day were not included in the study.

Evaluations were performed before (less than 1 month before the procedure), and 3, 6 and 12 months after the procedures. Post-surgery data collection was coordinated with the participants’ scheduled surgery follow-up appointments. Before procedures all patients were suggested to stop smoking, but none had specific smoking cessation program.

Participants provided informed written consent and the study was approved by the Institutional Review Board of Monaldi, S Maria della Pietà, Casoria, Hospital, Naples.

### Smoking status

The following questionnaires were administered: 1. Baseline on smoking habit: i. Smoking initiation age; ii. Duration of smoking; iii. Number of cigarettes smoked per day; and iv. Number of attempted cessations); 2. The Fagerström test on smoking dependence, and 3. the follow-up questionnaire: the daily cigarette smoking, and reasons for quitting; 3. Follow-up questionnaire: i. Number of cigarettes smoked per day; ii Reason for smoking quit (only for quitters).

### Statistics

Descriptive statistics, means and standard deviation (SD) were calculated for all quantitative variables, while percentages were generated for qualitative variables. Distributions were defined by the Kolmogorov-Smirnov test. Categorical variables were compared by bivariate statistical analysis with Chi-square test.

For parametric data (e.g., BMI, age), comparisons between continuous variables were done by ANOVA and Student’s *t*-test for paired or unpaired data. For non parametric data (number of smoking cigarettes and Fagerström score), Kruskal-Wallis one-way analysis of variance and Wilcoxon test were used.

The Pearson's correlation coefficient or Spearman rank correlation coefficients were used to analyze correlations between continuous variables. The level of significance was set at *p* < 0.05 in all analyses. Statistical analyses were performed using GraphPAD Prism, version 4 (GraphPAD Inc, San Diego, CA, USA).

## Results

We studied seventy-eight patients (69 females) (Table 1). Twenty-eight patients underwent IB, 30 patients LAGB, and 20 surgical procedures (SPG) (5 patients laparoscopic gastric bypass, and 15 patients sleeve gastrectomy).

**Table 1:** Characteristics of smoker obese patients undergone intra-gastric balloon (IB), lap-band laparoscopic surgery (LAGB), and laparoscopic gastric bypass/sleeve gastrectomy surgery (SPG)

	IB	LAGB	SPG	*p*
Subject number	28	30	20	
Sex (female/male)	24/4	26/4	14/6	0.1
Age (years)	35.0 ± 7.7	34.1 ± 8.7	37.8 ± 6.1	0.25
BMI pre-intervention (kg/m^2^)	42.5 ± 4.0	42.5 ± 4.7	46.8 ± 4.3	0.012
Age at smoking initiation, years	17.1 ± 2.0	16.8 ± 3.7	16.9 ± 2.3	0.42
Duration of smoking, years	16.0 ± 5.7	15.4 ± 6.9	17.7 ± 6.3	0.56
Number of cigarettes smoked per day*	20 (10–30)	20 (10–30)	20 (15–25)	0.79
Number of smoking cessation attempts*	0 (0–2)	0 (0–3)	0.5 (0–4)	0.38
FTND, score*	3 (0–8)	4 (0–8)	4 (0–8)	0.46

FTND: Fagerström Test for Nicotine Dependence

Values are expressed as mean ± SD except otherwise specified. * = median (range)

Table 1 shows the cigarette smoking habits in all groups before the procedures. We found no differences in daily cigarette smoking, smoking behavior/cessation and pack/year among all the three groups (always *p* > 0.05). Similarly, no differences in smoking dependence as calculated by Fagerström test were found among groups (*p* = 0.26).

In the IB, LAGB and SPG the BMI of obese patients was significantly reduced after 3, 6 and 12 months following the procedures (always *p* < 0.001) (Table 2).

**Table 2:** Body mass index and number of cigarettes smoked per day in persistent smoker patients undergone intra-gastric balloon (IB), lap-band laparoscopic surgery (LAGB), and laparoscopic gastric bypass/sleeve gastrectomy surgery (SPG) at 3, 6 and 12 months

	IB	LAGB	SPG	*p*
BMI at 3 m (kg/m^2^)	38.4 ± 3.1	37.0 ± 3.3	40.0 ± 3.6	0.009
BMI at 6 m (kg/m^2^)	35.9 ± 3.2	33.2 ± 2.8	35.7 ± 3.4	0.02
BMI at 12 m (kg/m^2^)	35.2 ± 2.9	32.7 ± 2.5	33.0 ± 3.6	0.01
Number of cigarettes smoked per day 3 m	18.4 ± 8.5	18.8 ± 7.2	19.0 ± 6.6	0.19
Number of cigarettes smoked per day 6 m	18.3 ± 8.6	18.6 ± 6.7	19.9 ± 6.8	0.95
Number of cigarettes smoked per day 12 m	18.7 ± 7.5	18.4 ± 6.4	18.0 ± 5.7	0.94

Values expressed as mean ± SD. 3 m = 3 months; 6 m = 6 months; 12 m = 12 months

**Fig. 1:**
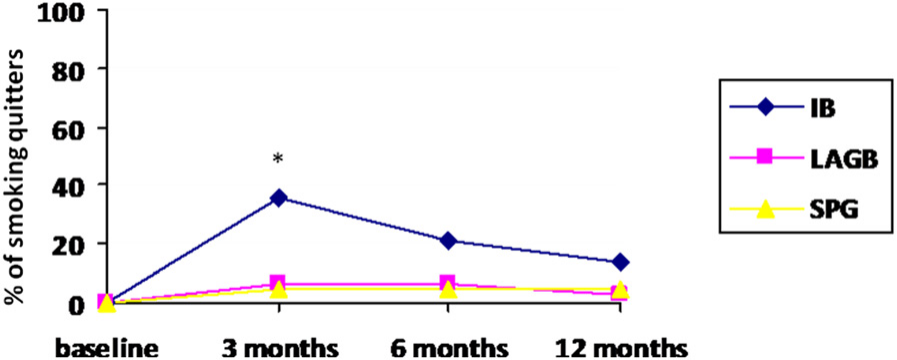
Percentage of smoking quitters who underwent intragastric ballon (IB), lap–band laparoscopic surgery (LAGB) and surgical procedures (SPG) at baseline and after 3, 6 and 12 months. * *p* = 0.02

After 3 months, the IB group showed a quitting rate higher than LAGB and SPG groups (36 %, 6 % and 5 %, respectively; *p* = 0.02) (Figure 1). No differences were found among the three groups at 6 months as well as 12 months after the procedures (IB 21 %, LAGB 6 %, and SPG 5 %; and IB 14 %, LAGB 3 %, SPG 5 %, respectively) (Fig. 1).

After three months, most of the IB group quitters (7 patients) associated their quitting to nausea and vomit suffered during the post-intervention period, while 3 reported personal reasons. In the LAGB and in the SPG the patients referred to have stopped smoking for personal reasons. No difference was observed for persistent smokers in the number of daily smoked cigarettes after one year in all groups (always *p* > 0.05) (Table 2). No correlation between BMI or weight loss and the number of smoked cigarettes was found.

No differences in weight lost between smokers and non-smokers after bariatric surgery were observed.

## Discussion

We have prospectively investigated the smoking status in obese patients before and after bariatric both invasive (such as gastric banding and bypass) and less invasive procedures (such as IB).

We have shown that the weight loss after bariatric procedures in obese patients was not associated with a significant reduction or stopping in cigarette smoking.

Our results on smoking status in obese patients after surgical procedures are in agreement with previous studies showing no differences between pre- and post surgery [6, 8]. In particular, Conason *et al.* in a prospective study on smoking indicated that rates do not notably change pre- or post- surgery [8]. Adams *et al.* have shown that in obese patient candidates to bariatric surgery, the patients who quit smoking within 6 months before surgery resumed after surgery [7].

Interestingly, in the first three months after the procedures, differently from surgical procedures, weight loss in the IB group was associated with a reduction or stopping in cigarette smoking. This result is quite surprising because an average weight gains usually occurs when people stop smoking [9, 10]. As similar weight loss was attained after bariatric surgery, it is unlikely that the higher frequency of stopping smoking in IB could depend on differences in BMI. Furthermore, we could exclude that surgical interventions itself have caused a greater tendency to stop as there were no differences in all three groups of patients after 6 and 12 months .

A causal mechanism of stopping smoking in obese subjected to IB may only be speculative, but the involvement of gastric distension induced by balloon is likely. Actually, smoke delays gastric emptying of solids, an effect not modulated by nicotine, and may enhance the side effects of nausea and vomit usually occurring after IB procedure. Furthermore, although it has not been assessed, we can exclude that our obese patients presented a low nicotine dependence. Indeed, it is now appreciated that the nicotine dependence for obese smokers are similar to those of highly dependent smokers [11]. Indeed, this case–control study was conducted in severe obese with moderate to heavy smoking exposure, with subjects not targeted by specific smoking cessation interventions, including counseling and/or pharmacotherapy.

Our study presents several limitations. First, it was based on a questionnaire and not on a direct measurement of smoke metabolites such as urine cotinine, screen or breath CO assessment. Secondly, we have studied only a limited number of subjects. However, ours was a single center study; we are aware that a multi-centric study with more patients would have made possible to detect significant differences among groups.

Both obesity and cigarette smoking carry an independent health risk, and the presence of both conditions may have a synergistic effect. However, little is known about the best treatment for people who smoke and are obese [12, 13]. Furthermore, as recently remarked, there is a lack of information about approaches to reduce tobacco use in populations that are particularly vulnerable or where tobacco has a disproportionately adverse effect, including people who have co-occurring conditions [3].

Although recent ASMBS guidelines recommend to avoid tobacco at all times by all obese patients before and after bariatric surgery given the increased risk for of poor wound healing and overall impaired health [14], no smoking cessation interventions, including counseling and/or pharmacotherapy have been indicated.
